# AO type C ankle fractures with additional posterior malleolus fragment: radiological and functional results of osseous reconstruction of the tibiofibular syndesmosis

**DOI:** 10.1007/s00590-026-04975-9

**Published:** 2026-10-09

**Authors:** Adriaan Laurens Franx, Floortje Huizing, Samuel Marinus Verhage, Pieta Krijnen, Inger Birgitta Schipper, Jochem Maarten Hoogendoorn

**Affiliations:** 1 Department of Trauma Surgery, Leiden University Medical Center, Leiden, Netherlands; 2 Department of Trauma Surgery, Haaglanden Medical Center, The Hague, Netherlands

**Keywords:** Trimalleolaor ankle fracture, Syndesmosis reconstruction, Syndesmotic stabilization, Weber C, Posterior malleolus, Posterior malleolar fracture

## Abstract

**Purpose:**

This study compares the syndesmosis congruity and functional outcomes after syndesmotic reconstruction by open reduction and internal fixation (ORIF) of posterior malleolar fractures (PMFs) sized  > 5% versus no fixation in type C ankle fractures.

**Methods:**

This multicenter prospective cohort study compared ORIF of PMFs (FIX) with no fixation (NO-FIX). Both groups underwent fibular and medial malleolus fixation according to AO principles, with trans-syndesmotic stabilization screws in case of instability. The primary outcome was syndesmotic alignment assessed on post-operative bilateral CT scans. The main secondary outcome measures were patient-reported functional outcome up to 1 year assessed with AAOS and OMAS questionnaires, and complications.

**Results:**

26 patients (17 FIX, 9 NO-FIX) were included, with a median PMF size of 20%. Syndesmotic stabilization with fib-tib screws was used in 6% of FIX vs 44% of NO-FIX (*p* = 0.03). Anatomical reduction (primary outcome) was achieved in 85% (FIX) versus 29% (NO-FIX, *p* = 0.02). AAOS and OMAS scores during follow-up were superior in FIX (linear mixed models, *p* < 0.01). The complication rate was higher in NO-FIX (55% vs. 0%, *p *< 0.001).

**Conclusion:**

in this study, ORIF of the PMF resulted in a higher rate of anatomically reduced syndesmosis, lesser need for syndesmotic stabilization screws, better functional outcome and fewer complications compared to no fixation. Fixation of PMFs in suprasyndesmotic fibula fractures with PMFs > 5% may be valuable, but more studies are needed to confirm this hypothesis due to study limitations.

## Background

In AO type C ankle fractures, injury of the tibiofibular syndesmosis is common. Anatomical repair and reduction of the syndesmosis is essential to prevent diastasis in the ankle-joint, since this is related to worse functional outcome and development of posttraumatic osteoarthritis (OA) in the ankle [1–3]. Repair may be performed by a closed or percutaneous reduction followed by stabilization with trans-syndesmotic screw placement. However, malreduction rates up to 52% are reported after placement of trans-syndesmotic screws [4].

Alternatively, in fractures with an additional posterior malleolar fracture, syndesmotic stability can also be achieved by open reduction and internal fixation (ORIF) of the posterior malleolar fragment (PMF) [5–8]. Anatomic reduction of the PMF provides ligamentotaxis of the posterior inferior tibiofibular ligament (PITFL), which is the most important contributor to the stability of the syndesmosis, resulting in anatomical reduction of the fibula into the incisura [5, 9, 10]. In addition to preserving natural mobility, PMF fixation may restore greater stability to the syndesmosis compared to using trans-syndesmotic screws alone [5]. Therefore, reconstruction of the incisura fibularis by fixation of the posterior malleolar fragment, regardless of its size may reduce the need for syndesmotic positioning screws [6].

This study aims to evaluate congruity and stability of the syndesmosis and functional outcome after ORIF of the PMF versus no fixation of the fragment in AO type C ankle fractures. We hypothesized that ORIF of the PMF would result in a higher rate of anatomically reduced syndesmosis, a lower rate of residual syndesmosis instability requiring trans-syndesmotic stabilization screws, reduced rates of osteoarthritis, and superior short-term patient-reported outcome scores compared to not fixing the PMF.

## Methods/design

### Study design and setting

This multicenter comparative observational study was conducted in Haaglanden Medical Center and Leiden University Medical Center, both level-1 trauma centers and teaching hospitals in The Netherlands. This study is registered at clinicaltrials.gov under protocol number NCT02599285. Ethical approval was obtained (see Declarations section).

### Study population

Between November 2015 and July 2021, all patients presenting with a trimalleolar suprasyndesmotic fibula fracture (AO type 44-C1, 44-C2, 44-C3) with additional PMF (> 5% of the involved articular surface, Table 1) between the age of 18 and 70 were eligible for inclusion. Patients were excluded in case of severe trauma with an Injury Severity Score  > 15 or multiple fractures, pre-existent disability or mobility problems, history of ankle fracture on the same ankle, inability to follow-up or insufficient understanding of the Dutch language.

**Table 1:** Overview of methods of radiologic measurements

Measurement	Radiologic modality	Description
*Anterior width*	CT-scan, axial view	Distance between most anterior point of incisura fibularis and nearest point of fibula
*Middle width*	CT-scan, axial view	Distance between subjective middle point of incisura fibularis and nearest point of fibula
*Posterior width*	CT-scan, axial view	Distance between most posterior point of incisura fibularis and nearest point of fibula
*Fibular rotation*	CT-scan, axial view	Angle of line between most posterior and most anterior point of incisura fibularis and line of fibular orientation, determined as line between most anterior and posterior point of fibula
*Tibiofibular clear space*	Radiograph, Mortise view	Horizontal distance between tibia and fibula at the level of the epiphyseal scar (or 10 mm above tibial plafond) on distal tibia
*Superior clear space*	Radiograph, Mortise view	Distance between tibial articular surface and talus
*Medial clear space*	Radiograph, Mortise view	Distance between talus and distal fibula, evaluated 5 mm below the talar dome
*Size posterior fragment*	Radiograph, lateral view/CT-scan sagittal view	Percentage of articular surface in sagittal diameter of tibial articular surface. (Size posterior fragment)/(size posterior fragment + size intact tibial articular surface)
*Step-off posterior fragment*	CT-scan, sagittal view	Distance of vertical displacement between the intact tibial plafond and the posterior fragment. On CT-scan, the slide showing the largest step-off was used

### Recruitment, informed consent and treatment

All patients presenting with ankle fractures at the participating Emergency Departments were screened for eligibility. Those who met the inclusion criteria provided written consent to participate. All participants in both treatment groups underwent a preoperative CT-scan to assess the size of the PMF. Patients were treated according to the standard treatment protocol in each hospital. In case of initial presentation in Leiden University Medical Center (LUMC), the PMF was not fixed (NO-FIX). These patients were positioned supine, and the lateral malleolus was fixed with one or two lag screws and/or a lateral plate. Proximal fibular fractures were not fixed. The medial malleolus was fixed with one or two cancellous screws. Subsequently, syndesmotic stability was assessed through intra-operative stress testing, using either the hook test or external rotational stress under fluoroscopic guidance. In cases of instability, the syndesmosis was reduced with a large syndesmosis clamp applied to the fibular ridge and the medial tibia at the level of the ankle joint. The reduction was evaluated in Mortise view using Weber’s indexes: equal joint space in the medial, superior, and lateral clear areas; a continuous ‘Shenton’s line’ with ‘Weber’s nose’ pointing to the tibial subchondral bone; and an uninterrupted line from the lateral articular surface of the talus to the distal fibula recess. The fibula’s position in the anterior–posterior direction relative to the tibia was also checked in lateral view. Finally, one or two (depending on the surgeon’s preference) trans-syndesmotic positioning screws were placed.

In Haaglanden Medical Center (HMC), ORIF of the posterior fragment (FIX) was performed using a posterolateral approach in prone position [11, 12]. The posterior fragment was fixed with lag screws or a buttress or antiglide plate. If possible, distal fibular fractures were addressed through the same incision. The medial malleolus was fixed similarly to the NO-FIX group. After fixation, the syndesmosis was tested in the same manner as in the NO-FIX group. Trans-syndesmotic positioning screws were placed only if the syndesmosis was unstable.

Postoperative treatment was identical for both groups. A cast was applied at the surgeon’s discretion, with daily low-molecular weight heparin given during immobilization. Patients were non-weight-bearing for 6 weeks, after which progressive weight-bearing was allowed. Trans-syndesmotic screw removal was not standardized, but followed participating hospital local procedures, after at least 8 weeks.

### Outcomes and follow-up

#### Radiologic outcome

The primary outcome was anatomical reduction of the syndesmosis assessed on bilateral postoperative CT-scan of the ankles. Reduction was assessed by the extent of tibiofibular diastasis and fibular rotation, as presented in Table 1. Various authors have attempted to define the range of normal tibiofibular relations [8, 13–15]. However, since interobserver variability is high and anatomic variation across humans is common, most values are of little clinical value [15–17]. A comparison of the contralateral ankle within the same patient provides more valuable information. Parameters most widely used in the literature to assess syndesmotic (mal)alignment are fibular rotation and syndesmotic diastasis measured on the most anterior (anterior width: AW), most posterior (posterior width: PW) and middle (middle width: MW) point of the incisura fibularis [18, 19]. In the sample size calculation of the original study protocol (see Sect. "Statistical analysis".), the syndesmosis was considered non-anatomical in case of a difference  > 1 mm in AW, PW or MW. However, during the inclusion period, recent literature suggested that a syndesmotic width difference of  > 2 mm more accurately assesses alignment. We decided to adopt this value, which rendered the original sample size calculation inaccurate. In the revised primary outcome, the syndesmosis is considered incongruent if there is a difference in AW, PW, or MW of  > 2 mm or fibular rotation >10 degrees compared to the uninjured contralateral ankle, as measured on the axial CT-scan 1 cm proximal to the tibial plafond [18, 19].

Secondary radiologic outcomes included quality of reduction of the PMF evaluated using step-off measured on sagittal CT-scan and extent of osteoarthritis after 52 weeks measured on plain radiographs. Step-off  > 1 mm was regarded as non-anatomical, since this is an independent risk factor for osteoarthritis [20]. Osteoarthritis was quantified using the Kellgren and Lawrence Score, modified by Kijowski et al. [21].

#### Clinical outcomes and patient-reported outcomes

Secondary clinical outcomes were fixation type, syndesmotic screw placement and length of hospital stay. All secondary interventions and complications were recorded. Secondary patient-reported outcomes included functional outcome assessed using the American Academy of Orthopaedic Surgeons foot and ankle Score (AAOS) and the Olerud and Molander Ankle Score (OMAS), both ranging from 0 to 100. A difference of 10 points on the AAOS was regarded to be clinically relevant (MCID) [22]. For OMAS, MCID’s of 7.5–15 are reported, in this study we adopted a MCID of 10. Perceived general health (EQ VAS for quality of life) and pain were both measured using a visual analogue scale (VAS) ranging from 0 to 100. The OMAS and VAS for pain and general health were scored at 6, 12, 26 and 52 weeks, and the AAOS at week 26 and 52.

### Sample size

A sample size calculation was conducted based on the primary outcome: anatomical reduction of the distal tibiofibular syndesmosis. Malreduction was defined as  > 1 mm difference in syndesmosis width compared to the contralateral ankle. Assuming a mean syndesmosis width of 4 mm (SD 1.19 mm), with 5% significance and 80% power, and accounting for a 15% dropout rate, 27 patients per group were needed [15].

### Statistical analysis

Baseline characteristics of participants are described using summary statistics. Continuous outcome measures are reported as mean and standard deviation, or median and range in case of skewed distribution, and were compared between the treatment groups by an unpaired t-test or Mann–Whitney test respectively. Categorical data are reported as number and frequency and were compared using a Chi-squared test or Fisher’s exact test. For clinical outcomes with a univariately significant association with fixation of the PMF, multivariable linear or logistic regression analysis was performed with adjustment for potential confounders. Linear mixed models (F-test) were used to compare the longitudinal scores on the AAOS, OMAS, VAS-pain and VAS-quality of life of the two groups during the follow-up, with adjustment for potential confounders. Baseline characteristics with a borderline univariate association with fixation of the PMF (*p* < 0.10) were considered potential confounders. Data was analyzed using the “Statistical Package for the Sciences” version 29.0. Statistical testing was 2-tailed and a *p*-value  < 0.05 was used as threshold for statistical significance. Baseline characteristics were complete for all cases. Missing values for outcome measures were not imputed.

## Results

### Patient characteristics

Between November 2015 and July 2021, 59 patients with AO type C ankle fractures were screened for eligibility. Of these, 24 were excluded based on inclusion criteria and 9 refused to participate, resulting in 17 patients in FIX and 9 in NO-FIX. Five FIX patients and two NO-FIX patients were lost to follow-up at 52 weeks. Baseline patient characteristics are presented in Table 2. Concomitant medial malleolus fractures were more common in FIX (35% vs. 89%; *p* = 0.01). In both groups, PMFs were mainly classified as Haraguchi types 1 and 2 or Bartonicek types 3 and 4. The median size of the posterior fragment on CT-scan was 20% for FIX and 14% for NO-FIX (*p* = 0.08). PMF comminution was present in 88% in FIX group and 89% in NO-FIX (*p* = 1.00).

**Table 2:** Patient demographics and clinical characteristics

	FIX		NO-FIX		*P*-value
n	17		9		
Female, n (%)	9	(53)	5	(56)	1.00
Age at trauma in years, mean (SD)	40	(17)	47	(14)	0.27
BMI, mean (SD)	28	(4)	29	(6)	0.61
*AO classification, n (%)*					
44-C1.3	5	(29)	5	(56)	0.43
44-C2.3	9	(53)	3	(33)	
44-C3.3	3	(18)	1	(11)	
*Bartonicek classification, n (%)*					
1	0	(0)	0	(0)	0.65
2	7	(41)	5	(56)	
3	9	(53)	4	(44)	
4	1	(6)	0	(0)	
*Haraguchi classification, n (%)*					
I	8	(47)	5	(56)	0.29
II	9	(53)	3	(44)	
III	0	(0)	1	(0)	
*Radiograph: preoperative*					
PMF size in %, median (range)	16	(10–48)	15	(8–24)	0.40
Medial fracture, n, (%)	6	(35)	8	(89)	**0.01**
*CT-scan: preoperative*					
PMF size in %, median (range)	20	(10–43)	14	(10–28)	0.22
Chaput-Tillaux fragment, n (%)	4	(24)	3	(33)	0.66
Wagstaffe-Le Fort fragment, n (%)	2	(12)	0	(0)	0.53
Comminution PMF, n (%)	15	(88)	8	(89)	1.00

PMF: posterior fragment; FIX: fixation of PMF; NO-FIX: no fixation of PMF. p-value < 0.05 was used as threshold for statistical significance.

### Radiologic outcome

**Fig. 1:**
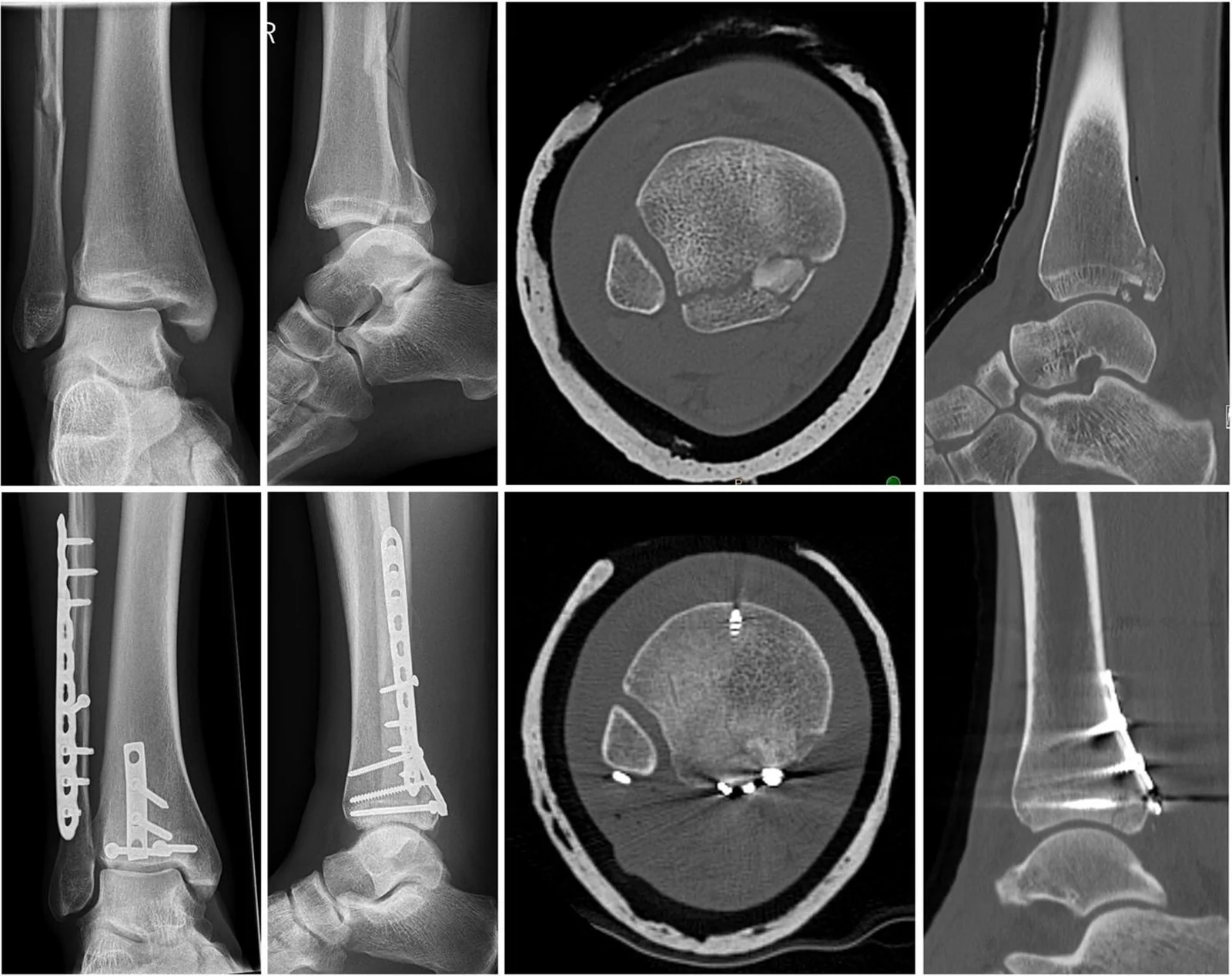
Preoperative (top row) and postoperative imaging (bottom row) within the same patient after ORIF of the fibula and posterior malleolus

**Fig. 2:**
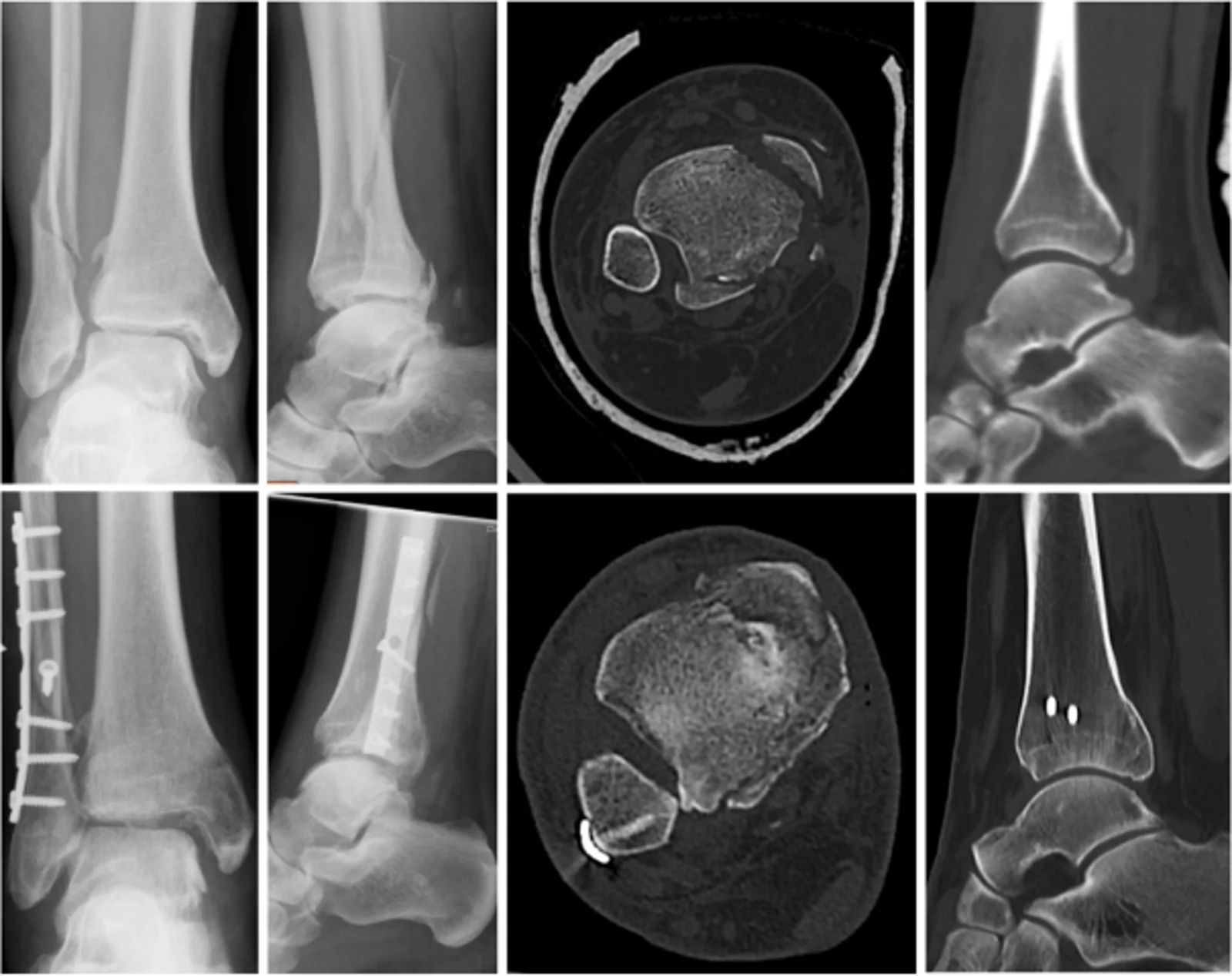
Preoperative (top row) and postoperative (bottom row) imaging within the same patient after ORIF of the fibula without fixation of the posterior malleolus

The analysis of the primary outcome (anatomical syndesmosis) included twenty patients with bilateral postoperative CT scans. In six patients (4 FIX, 2 NO-FIX), only unilateral CT scans were available. Assessed on bilateral postoperative CT-scan, the syndesmosis was regarded anatomical in 85% in the FIX group versus 29% in the NO-FIX group (*p* = 0.02; Table 3; Figs. 1 and 2). The unadjusted odds ratio [OR] of anatomical syndesmosis for FIX vs. NO-FIX was 13.75 (95% confidence interval [CI] 1.48–127; *p* = 0.02). After adjustment for concomitant medial malleolus fracture, this association was borderline significant (OR 12.49; 95% CI 0.93–169; *p *= 0.06). PMFs were more often anatomically reduced in the FIX group: 59% versus 11% (*p* = 0.04; unadjusted OR 11.43; 95% CI 1.16–133). After adjustment for concomitant medial malleolus fracture, this association remained statistically significant (OR 15.98; 95% CI 1.19–215; *p* = 0.04). Mean postoperative PMF step-off was 0.8 mm versus 2.7 mm (*p* < 0.001) for FIX and NO-FIX respectively. This difference remained statistically significant after adjustment for concomitant medial malleolus fracture (mean difference 1.9 mm; 95% CI 1.0–2.9; *p *< 0.001). At one year postoperatively, plain radiography was available for 17 patients (9 missing). Radiologic signs of post-traumatic OA ≥ grade 2 were present in 50% for NO-FIX and 27% for FIX (*p* = 0.60).

**Table 3:** Clinical and patient-reported outcomes after fixation or no fixation of the posterior fragment

	FIX (N = 17)		NO-FIX (N = 9)		unadjusted *p*-value
	N		N		
*Postoperative CT-scan, n (%)*					
Anatomical syndesmosis	13	11 (85)	7	2 (29)	**0.02**
Anatomical reduction PMF (step-off ≤1 mm)	17	10 (59)	9	1 (11)	**0.04**
PMF step-off in mm, mean (SD)	16	0.8 (0.9)	9	2.7 (1.0)	**< 0.001**
*Radiograph: week 52*					
Osteoarthritis ≥ grade 2, n (%)	11	3 (27)	6	3 (50)	0.60
*Functional outcome*					
*AAOS, median (range)*					
Week 26	12	85 (79–96)	4	71 (52–82)	**0.008**
Week 52	12	89 (66–99)	6	83 (47–91)	
*OMAS, median (range)*					**0.002**
Week 6	14	15 (5–55)	5	5 (0–30)	
Week 12	12	40 (15–80)	4	18 (10–30)	
Week 26	12	63 (30–85)	4	35 (15–50)	
Week 52	10	80 (45–95)	6	63 (15–75)	
*VAS-pain, median (range)*					**0.03**
Week 6	14	15 (0–60)	5	15 (10–80)	
Week 12	12	30 (0–70)	4	28 (10–75)	
Week 26	12	15 (0–30)	4	45 (10–80)	
Week 52	12	18 (5–45)	6	28 (0–80)	
*VAS-quality of life, median (range)*					0.35
Week 6	14	78 (60–90)	5	70 (50–95)	
Week 12	11	75 (55–100)	4	85 (70–95)	
Week 26	11	85 (65–100)	3	85 (65–95)	
Week 52	12	88 (75–100)	5	80 (50–90)	

PMF: posterior fragment; FIX: fixation of PMF; NO-FIX: no fixation of PMF. p-value  < 0.05 was used as threshold for statistical significance.

### Clinical outcome and patient reported outcomes

In the FIX group, a trans-syndesmotic screw was placed in one (6%) patient. In the NO-FIX group, one or two trans-syndesmotic screws were placed in four (44%) patients (*p* = 0.03; Table 4) due to instability during intraoperative stress testing. The median hospital length of stay was two days in both groups (*p* = 0.42). Complications were observed in zero (0%) versus four (45%) cases (*p* < 0.001) for FIX and NO-FIX respectively. Infection occurred in three cases in the NO-FIX group. In one of these infection cases secondary dislocation after removal of the syndesmotic screw also occurred, without the need for reoperation. One patient in NO-FIX underwent a secondary operation due to severe malreduction of the syndesmosis after syndesmotic screw placement, which was observed on postoperative CT-scan.

**Table 4:** Surgical techniques and complications regarding fixation of the posterior fragment

	FIX (N = 17)		NO-FIX (n = 9)		*p*-value
*Syndesmotic positioning screw, n (%)*	1	(6)	4	(44)	0.03
1 screw	1	(100)	3	(75)	
2 screws	0	(0)	1	(25)	
*Syndesmotic screw removal*	0	(0)	3	(75)	
External fixation, n (%)	2	(12)	2	(22)	0.59
*Hospital stay in days, median (range)*	2	(1–5)	2	(1–5)	0.46
*Complication, n (%)*	0	(0)	4	(45)	**< 0.003**
Infection	0	(0)	3	(33)	
Secondary dislocation	0	(0)	1	(11)	
Secondary operation	0	(0)	1	(11)	
PE/DVT	0	(0)	0	(0)	
Other complication	0	(0)	0	(0)	

PMF: posterior fragment; FIX: fixation of PMF; NO-FIX: no fixation of PMF; PE: pulmonary embolism; DVT: deep venous thrombosis. p-value < 0.05 was used as threshold for statistical significance.

Patient-reported outcomes are presented in Table 3. In total, 73% of questionnaires at week 52 were available. In the FIX group, superior AAOS scores (adjusted for concomitant medial malleolus fracture; F-test, *p* = 0.007) and superior OMAS scores (adjusted for concomitant medial malleolus fracture; F-test, *p* = 0.005) during follow-up were observed. Pain scores during follow-up were significantly lower in the FIX group (adjusted for concomitant medial malleolus fracture; F-test, *p* = 0.01). Perceived general health was comparable between the groups during follow-up (adjusted for concomitant medial malleolus fracture; F-test, *p* = 0.09).

## Discussion

This study aimed to evaluate congruity and stability of the syndesmosis and functional outcome after open reduction and internal fixation of the PMF versus no fixation of the fragment in AO type C ankle fractures with additional PMF. PMF fixation yielded superior rates of anatomical reduced syndesmosis and PMF’s, with fewer cases of instability requiring syndesmotic stabilization screws. No difference in 1-year posttraumatic osteoarthritis rates was found. However, considering the relatively short follow-up period, data regarding posttraumatic osteoarthritis should be interpreted with caution. Differences in AAOS, OMAS and pain scores during follow-up were statistically significant and clinically relevant, favoring PMF fixation.

Our findings are in line with other publications. In a retrospective study, Miller et al. found comparable functional outcomes and syndesmotic reduction rates between PMF fixation and syndesmotic screws [6]. The authors recommended anatomical reconstruction of the incisura fibularis by PMF fixation, regardless of size, as this may eliminate the need for syndesmotic screws. Baumbach et al. retrospectively reviewed 236 trimalleolar ankle fractures and concluded that ORIF of the PMF significantly reduced the need for trans-syndesmotic fixation (25%) compared to closed reduction and fixation (61%) or no fixation (63%) [23]. They concluded that ORIF for all PMFs, regardless of size, more effectively restores syndesmotic stability than leaving the PMF unfixed or using closed reduction. Caglar et al. retrospectively studied 94 lateral ankle fractures (including 28% classified as AO type C) with PMFs treated using buttress plate fixation via a posterolateral approach [7]. After lateral and posterior fixation, no trans-syndesmotic screws were required. Clinical outcomes were good or excellent in 92% of cases.

Various studies have shown that fibular malreduction in the incisura fibularis is common after placement of syndesmotic screws, whether via closed or open reduction [4, 24]. The posterior incisura acts as a buttress, preventing the fibula from rotating out of the posterior part of the articulation, which is difficult to appreciate on radiographs [4, 13, 25]. Gardner et al. evaluated reduction on postoperative CT-scans and observed fibular malreduction in 52% of patients treated with syndesmotic screws [4].

Moreover, syndesmotic screws are associated with morbidity. Syndesmotic screws are routinely removed in many clinics, causing the patient to undergo a second surgical procedure, which has its own inherent costs, risks and burdening. In contrast, PMF fixation reduces the need for syndesmosis implants, thereby minimizing the risk for these issues.

Traditionally, PMF size has been the primary indication for fixation, with fragments involving 25–33% of the articular surface generally considered the threshold for surgical treatment. However, accumulating evidence suggests that treatment decisions should be guided by factors beyond fragment size, including PMF morphology, fragment displacement, syndesmotic stability, articular impaction, and the presence of intercalary fragments [26–29]. In particular, medial extension-type fragments (Haraguchi type II or Bartoníček type 3) have been associated with poorer clinical outcomes than isolated posterolateral fragments [29]. Consequently, current concepts advocate an individualized treatment strategy rather than relying on fixed thresholds for fragment size or displacement [26–28]. This approach is consistent with the rationale underlying the present study. Given that syndesmotic disruption is highly prevalent in AO type C ankle fractures, we believe that the threshold for PMF fixation should be low when fixation is expected to contribute to restoration of syndesmotic stability, irrespective of fragment size.

The strengths of this study include the use of both pre- and postoperative bilateral CT-scans for radiologic evaluation and the OMAS and AAOS for functional assessment. However, this study has definite limitations. Treatment allocation was not randomized but determined by the protocol of the participating hospital. Consequently, the comparison reflects not only two treatment strategies but also two different centers and surgical teams, introducing potential center-related bias and residual confounding. Furthermore, postoperative management was not standardized across centers. Decisions regarding cast immobilization and the removal of syndesmotic screws were left to the discretion of the treating surgeon. Although most syndesmotic screws were routinely removed after approximately 8 weeks, some were retained or removed only if clinically indicated. These variations in postoperative care may have influenced the observed outcomes and limit the generalizability of the findings, particularly as on-demand removal of syndesmotic screws has become increasingly accepted following the publication of a randomized controlled trial demonstrating its safety and effectiveness [30]. Unfortunately, the number of patients meeting the inclusion criteria was lower than expected, leading to a long recruitment period. Eventually, the study was stopped before the intended number of patients was included, leading to an underpowered analysis for the primary outcome. During this long recruitment period, changes in surgical techniques or surgeon experience may have occurred. On the other hand, statistically significant differences in other outcome measures such as functional outcome were observed even in this small sample, reflecting the large difference in clinical outcomes after the two treatment modalities. Not all NO-FIX patients received trans-syndesmotic screws, making it difficult to isolate their effects. Radiological measurements lacked interobserver agreement analysis, and some cases had only unilateral CT-scans. Moreover, as follow-up was only 1 year, data regarding development of posttraumatic osteoarthritis and potential functional implications should be interpreted with caution.

Finally, the extended inclusion period should be considered when interpreting the findings, as both surgical techniques and the understanding of syndesmotic injuries have evolved over time. In the present study, all patients requiring syndesmotic stabilization were treated with trans-syndesmotic screw fixation. However, flexible suture button fixation has since become increasingly adopted in clinical practice, which may limit the generalizability of these findings as such flexible fixation methods were not used in this study. A meta-analysis of randomized controlled trials demonstrated superior functional outcomes and lower complication rates following suture button fixation compared with syndesmotic screw fixation for syndesmotic injuries [31]. In addition, radiographic assessment of the tibiofibular syndesmosis remains controversial, as conventional radiographs are known to have limited reliability in detecting syndesmotic malreduction [13, 32]. Emerging techniques, including intraoperative three-dimensional imaging and (needle) arthroscopy, are increasingly being studies for the assessment of syndesmotic integrity and reduction [33–36]. Although the clinical value of these modalities has yet to be fully established, their incorporation into future studies may provide a more accurate evaluation of syndesmotic reduction and help further define the role of posterior malleolar fragment fixation in restoring syndesmotic stability.

## Conclusions

Despite the study’s limitations, the data suggest that ORIF of the PMF may result in higher rates of anatomically reduced syndesmosis with lower rates of persisting syndesmotic instability requiring stabilization screws, improved functional outcomes and fewer complications compared with no fixation of the PMF. These results support consideration of posterior malleolar fixation in AO type C ankle fractures with a posterior fragment involving more than 5% of the articular surface. If syndesmotic instability persists after fixation of the lateral, medial, and posterior malleolar fractures, trans-syndesmotic screw fixation should be considered. However, larger randomized clinical trials with longer follow-up are needed to confirm these findings and further value the role of PMF fixation.

## Data Availability

No datasets were generated or analysed during the current study.
